# Determinants of Non-paid Task Division in Gay-, Lesbian-, and Heterosexual-Parent Families With Infants Conceived Using Artificial Reproductive Techniques

**DOI:** 10.3389/fpsyg.2020.00914

**Published:** 2020-05-13

**Authors:** Loes Van Rijn - Van Gelderen, Kate Ellis-Davies, Marijke Huijzer-Engbrenghof, Terrence D. Jorgensen, Martine Gross, Alice Winstanley, Berengere Rubio, Olivier Vecho, Michael E. Lamb, Henny M. W. Bos

**Affiliations:** ^1^Preventive Youth Care, Research Institute of Child Development and Education, University of Amsterdam, Amsterdam, Netherlands; ^2^Department of Psychology, Swansea University, Swansea, United Kingdom; ^3^Research Institute of Child Development and Education, University of Amsterdam, Amsterdam, Netherlands; ^4^UMR 8216 Centre d’Etudes en Sciences Sociales du Religieux, Paris, France; ^5^Division of Social and Developmental Psychology, University of Cambridge, Cambridge, United Kingdom; ^6^Institut Français des Sciences et Technologies des Transports, de l’Aménagement et des Réseaux, Paris, France; ^7^Département de Sciences Psychologiques, Université Paris Nanterre, Nanterre, France

**Keywords:** non-paid task division, parenting, gay, lesbian, heterosexual, first-time parents, infants, determinants

## Abstract

**Background:** The division of non-paid labor in heterosexual parents in the West is usually still gender-based, with mothers taking on the majority of direct caregiving responsibilities. However, in same-sex couples, gender cannot be the deciding factor. Inspired by Feinberg’s ecological model of co-parenting, this study investigated whether infant temperament, parent factors (biological relatedness to child, psychological adjustment, parenting stress, and work status), and partner relationship quality explained how first-time gay, lesbian, and heterosexual parents divided labor (childcare and family decision-making) when their infants were 4 and 12 months old. We also tested whether family type acted as a moderator.

**Method:** Participants were drawn from the new parents study. Only those who provided information about their biological relatedness to their child (*N* = 263 parents) were included. When infants were 4 months (T1), parents completed a password-protected online questionnaire exploring their demographic characteristics including work status and standardized online-questionnaires on task division (childcare and family decision-making), infant temperament, parental anxiety, parental depression, parental stress, and partner relationship satisfaction. When infants were 12-months-old (T2), parents provided information about task division and their biological relatedness to their children.

**Results:** Linear mixed models showed that no factor explained the division of family decision making at T1 and T2. For relative time spent on childcare tasks at T1, biological relatedness mattered for lesbian mothers only: biologically related mothers appeared to spend more time on childcare tasks than did non-related mothers. Results showed that, regardless of family type, parents who were not working or were working part-time at T1 performed more childcare tasks at T1. This was still true at T2. The other factors did not significantly contribute to relative time spent on childcare tasks at T2.

**Conclusion:** We had the opportunity to analyze the division of non-paid tasks in families where parenting was necessarily planned and in which gender could not affect that division. Although Feinberg’s model of co-parenting suggests that various factors are related to task division, we found that paid work outside the home was most important during the first year of parenthood in determining caregiving roles.

## Introduction

During the transition to parenting, new parents need to make decisions about how parenting roles will be shared (Cao et al., 2016). Dissatisfaction with this division is a major source of parenting stress which undermines partner relationship satisfaction and parental well-being (Patterson, 1988) and which in turn might be related to how children fare (e.g., Stone et al., 2016). Since research on how parents divide and share co-parenting responsibilities and roles has mainly focused on heterosexual couples and their biological children, gender is often conflated with caregiving role. We thus know little about how parents decide caregiving roles when gender is the same for both parents, such as when same-sex parents use artificial reproductive techniques to conceive (Goldberg, 2010). In these families, only one parent is biologically related to the child. The present study focused on the division of non-paid tasks during the first year of parenthood within three different family types: gay–father families with infants who were conceived through surrogacy procedures, lesbian–mother families whose infant offspring were conceived by means of insemination with donor sperm (DI), and heterosexual-parent families whose infants were conceived through *in vitro fertilization* (IVF).

When looking at the division of non-paid tasks, three subgroups can be identified. The first group comprises household tasks including all the (non-paid) tasks that need to be done to maintain family members and/or a home (Coltrane, 2000) such as laundry, cooking, taking care of plants or yard, and car maintenance (Cowan and Cowan, 1988). Childcare comprises the second set of non-paid tasks and includes feeding, dressing, bathing, arranging for childcare or babysitting (Cowan and Cowan, 1988). The third group of non-paid tasks includes family decisions such as planning for vacations, deciding how to arrange finances (e.g., taxes, insurance), and deciding about community involvement (Cowan and Cowan, 1988).

After the birth of a child, parents need to divide both non-paid and paid tasks. Even though the participation of women in paid labor in Western societies has increased, different-sex parents’ division of non-paid parenting tasks has largely remained unequal, with women doing more of the non-paid tasks than men (Baxter et al., 2008, 2015; Bianchi et al., 2012). It is often assumed that this pattern can be explained by gendered roles (i.e., roles that are seen as appropriate to gender in accordance with prevailing cultural norms) and gender ideology (i.e., normative ideas about accepted roles and inherent features of human females and males) on a societal level (Geist, 2005; Greenstein, 2009; Nyman et al., 2013). Gender inequality persists and is represented through daily interactions ‘doing gender,’ which “involves a complex of socially guided perceptual, interactional, and micropolitical activities that cast particular pursuits as expressions of masculine and feminine nature” (West and Zimmerman, 1987, p. 126). An example of doing gender might involve women affirming their femininity by showing their competence as nurturers or household organizers or men affirming their masculinity by avoiding housework. Men may not incorporate the caregiver role into their self-concepts to the same extent as mothers (Hall et al., 1995), while some men also see their involvement in paid employment as an important contribution to the caregiving of the child (Chan et al., 1998).

This traditional pattern of the non-paid labor division in different-sex families does not appear susceptible to change when maternal education increases. Recent studies have demonstrated that while childless women often aspire to more equitable divisions of caregiving, after the transition to parenting both men and women revert to more traditional models of caregiving roles (Baxter et al., 2015). Thus, the division of non-paid labor is usually still gender-related (Goldberg and Perry-Jenkins, 2004). However, in same-sex couples, gender cannot be the deciding factor. Therefore, it may be revealing to investigate how same-sex families divide their non-paid tasks.

Previous research on same-sex parents has indicated that lesbian and gay couples share household and childcare tasks in a more egalitarian way than heterosexual couples do (e.g., Vecho et al., 2011; Goldberg et al., 2012; Farr and Patterson, 2013). However, heterogeneity exists *within* same-sex families with regard to their division of household- and childcare tasks (i.e., not all families report an egalitarian division; Tornello et al., 2015) and thus it is valuable to investigate how these differences come about. With the exception of one study with lesbian-mother families (Goldberg and Perry-Jenkins, 2007), no studies on this topic have focused on the first year of parenthood. That is surprising, because most transitions in parenthood are made in this period (Durtschi et al., 2017) when co-parent relationships are developed (Van Egeren, 2004). Since infancy provides a valuable period to gain insight into how parents divide their paid and non-paid tasks, we decided to focus on the division of tasks by same-sex and different-sex parents with infants.

Feinberg (2003) provides a helpful model for determining which factors could influence the way parents divide their non-paid tasks within their families during the first year of their children’s lives. In this ecological model of co-parenting, co-parenting consists of four components (support/undermining, childrearing agreement, division of labor, and joint family management). These components do not function on their own but are directly and indirectly influenced by the child, parent, and interpersonal factors. Therefore, we sought to investigate which child (i.e., infant temperament), individual parent (i.e., biological relatedness to child, gender, work status, psychological adjustment, parenting stress), and interparental factors (i.e., partner relationship quality) explained how first-time gay, lesbian, and heterosexual parents divided labor when their infants were 4- and 12-months-old.

The link between infant temperament and non-paid task division is emphasized in family systems theory which argues that systems within the family are interdependent (Minuchin, 1985) and thus that it is important to examine the possible link between infant temperament and task division. In general, infant temperament (i.e., biologically based individual differences in reactivity and the ability to self-regulate; Rothbart and Bates, 1998) influences the way parents feel and act. For example, parents with highly irritable infants experience more parenting stress than parents with less irritable infants (Mulsow et al., 2004). Another study showed that parents of infants who are easily distressed, fearful, and sad reported higher levels of depressive symptoms and stress, and lower parental efficacy than parents with infants who had more positive temperaments (Solmeyer and Feinberg, 2011). However, associations between child temperament and co-parenting are not consistently found. Some researchers found no evidence for direct relations (e.g., McHale et al., 2004) while others did (e.g., Burney and Leerkes, 2010). For task division specifically, Burney and Leerkes (2010) found that for mothers (but not fathers), infants’ distress to novelty at 6 months was negatively related to a sum score of three aspects of task division, including parents’ perception of their partners as doing more childcare tasks, satisfaction with how they were sharing parenting tasks, and whether the division met their prior expectations.

One of the parent factors we studied was biological relatedness. Social structural theory (Eagly and Wood, 1999) argues that “the roles people occupy – which may be due to individual choice, sociocultural pressures, or biological potentials – lead them to develop psychological qualities and, in turn, behavior to fit those roles” (Katz-Wise et al., 2010, p. 2). Biological factors such as experiencing pregnancy, giving birth and being able to breastfeed are thought to increase the time spent in childcare. This has indeed been supported by empirical research on families with infants showing that fathers participate less in childcare when mothers are breastfeeding (Gamble and Morse, 1993; Earle, 2000). In addition, the only study on task division by lesbian couples with infants showed that biological mothers tended to spend more time on childcare than non-biological mothers when their children were 3 months old (Goldberg and Perry-Jenkins, 2007). However, studies on lesbian families with older children have reported mixed findings; some studies showed no differences between biological and non-biological mothers in time spent in childcare (Chan et al., 1998; Gartrell et al., 1999, 2000) while other studies found differences (Bos et al., 2007; Downing and Goldberg, 2011; Vecho et al., 2011). These varying findings may suggest that lesbian mothers have a more flexible caregiving role division with caregiving roles flexibly changing over time.

In addition, Hamilton’s (1964) theory of selection (also known as the theory of inclusive fitness) assumes that altruistic behavior in humans is adaptive when it increases the genetic fitness of individuals. Raising a child has economic, physical, and mental costs. Investment in these costs would be particularly efficient for parents who know that they share genetic material with a child. Thus, biologically related parents should invest more in their children than non-biological parents do because unrelated children offer few reproductive benefits to their parents, which make it less profitable for them to invest valuable resources. Extending this idea to same-sex families might mean that biological parents in same-sex families would spend more time in childcare than non-biological parents. The only study on the relation between gay fathers’ biological relatedness and division of labor found that the amount of household and childcare labor that men reported doing was unrelated to biological relatedness (Tornello et al., 2015). However, the age range of the children in this study of 52 gay men was very broad (0–12 years). We sought to determine whether results were the same in a study involving same-sex parents with young infants.

As a second parent factor, we focused on time spent on paid work outside the home as a possible determinant of non-paid task division. The time-constraint theory of Artis and Pavalko (2003) argued that there are only a finite number of hours in the day to perform unpaid and paid labor and, if one partner is working more outside the home, that partner has less time to participate in unpaid labor at home. Empirical evidence from studies among same-sex and different- sex parent families showed that partners who spent more time outside the home indeed spent less time doing household and childcare tasks (Downing and Goldberg, 2011; Goldberg et al., 2012; Tornello et al., 2015). In their study of different-sex families and lesbian-mother families, Patterson et al. (2004) found that the lesbian mothers spent the same number of hours in paid employment and were equally involved in childcare tasks. Within different-sex families, on the other hand, fathers spent twice as many hours in paid employment as did mothers, resulting in mothers being more intensively involved in childcare tasks than fathers. In contrast, a recent study of parental involvement (including perception of level of involvement in childcare and upbringing) by adoptive gay fathers with children between 1 and 9 years old showed no relation between parental involvement and number of hours devoted to paid work (Feuge et al., 2019). We investigated whether this was true in same-sex families with infants only.

It is also important to consider whether parental psychological wellbeing affects task division in the first year of parenthood (Feinberg, 2003). Even though the anticipation and the birth of a child are often associated with positive emotions, there is also the risk of developing psychological problems, such as depression (Gross and Marcussen, 2017) and anxiety (Heron et al., 2004). Empirical studies focusing on the division of non-paid tasks and parental psychological adjustment suggested that these concepts are related. For example, when the distribution of household tasks is experienced as fair, mothers display few symptoms of depression, but when it is perceived as unfair, mothers show more such symptoms (Glass and Fujimoto, 1994; Lennon and Rosenfield, 1994). However, these studies included parental wellbeing as an outcome variable rather than as a predictor. This study was the first to investigate whether parental psychological adjustment also predicted how same-sex and different-sex parents divide non-paid tasks.

The last individual parent factor investigated as a predictor was parenting stress (i.e., feelings of stress caused by the fact that parenting demands are higher than the personal and social resources available; Cooper et al., 2009). Mothers appear to experience more parenting stress than fathers do (Ostberg, 1998). Musick et al. (2016) found that this difference might be due to the difference in how fathers and mothers spend time with their children. Mothers performed more household- and childcare tasks, had a lower quality of sleep and less leisure time than did fathers, whereas fathers spent more time with the children in activities that were high in enjoyment and low in stress (e.g., play and leisure). Ehrenberg et al. (2001) also found that mothers in dual-earning families performed more childcare tasks than fathers. They suggested that mothers may feel the need to bear the greater responsibilities for taking care of their children to feel like “good” mothers (Ehrenberg et al., 2001). Perhaps this feeling contributes to higher feelings of parenting stress. We sought to explore these issues in same-sex families.

Parental relationship quality (an interparental factor) is often deemed the most important family factor influencing co-parenting relations (Feinberg, 2003). However, with regard to the division of childcare and household tasks it is known that perceptions of fairness about family work are often more related to relationship quality than the actual division of labor (Grote et al., 2002; Claffey and Mickelson, 2009): Parents rate their relationship quality more positively when they think that the family work has been distributed fairly. Ehrenberg et al. (2001), on the other hand, found that, even though mothers spent a significantly greater proportion of time on childcare tasks than fathers did, as long as both parents were equally involved in performing the “fun” tasks (e.g., planning and executing family outings together), both parents felt satisfied in their relationship. We explored whether there was a relation between relationship quality and the division of childcare and household tasks in same- and different–sex families with infants.

In sum, this study aimed to investigate whether child temperament, individual parent characteristics (i.e., biological relatedness to child, work status, psychological adjustment, parenting stress), and partner relationship quality explained how first-time gay, lesbian, and heterosexual parents divided labor (family decisions making and childcare) when their infants were 4 and 12 months old. In addition, we sought to investigate whether significant factors worked the same way in gay, lesbian, and heterosexual parents by testing whether family type acted as a moderator. In general, we hypothesized that all factors are related to non-paid task division. For two factors, based upon prior theoretical and empirical research, we had two specific hypotheses: (1) Parents who were biologically related to their children would spend more time on childcare tasks, and (2) Parents who spent more time working outside the home would spend less time on family decision making and childcare tasks.

## Materials and Methods

### Participants

The participants for the current study were drawn from the new parents study (NPS). The NPS sample (*N* = 140 families) consists of 38 gay-father families, 61 lesbian-mother families, and 41 heterosexual-parent families from the United Kingdom (23.6%), the Netherlands (33.6%), and France (42.9%). For the current study, data were only used when parents provided information about their biological relatedness to their child (answer possibilities were yes or no). Six gay couples from the United Kingdom and one gay couple from France did not provide biological relatedness information. In addition, in two lesbian couples from the United Kingdom and one lesbian couple from France, only one lesbian mother provided information about their biological relatedness. This led to an analytic sample of 263 parents from 133 families.

At the start of the study (T1; when infants were around 4 months old), the mean age of the parents in the analytic sample was 34.74 years (ages ranged from 22 to 59 years old). On average at T1, the parents had been together for 7.95 years (*SD* = 3.47) and most of them were married or in civil partnerships (79.5%). A small number of the parents lived in rural areas (6.5%), while the remaining parents lived in small- (33.5%), medium- (32.3%), or large-sized cities (27.8%). Most parents were highly educated (83.1% had obtained a college degree or higher) and their yearly income was above average: 69.7% earned over 42,365 US dollars per year. The majority of the parents worked full-time (62.4%). The majority of the British and Dutch parents were White (94.5%); we did not have permission to obtain information about the ethnic background of the French parents. Almost all parents (93.2%) experienced good to excellent health. Most parents had singletons (85.2%) and they had slightly more girls (59.7%) than boys. The mean age of the children was 3.32 months (*SD* = 0.61).

There were no significant differences between the family types with regard to parental ethnic identity and the infants’ gender (see Table 1). However, there were significant differences between gay fathers, lesbian mothers, and heterosexual parents with regard to parental age, relationship duration, having twins or singletons, working status, family income, and where families lived (residency).

**TABLE 1:** Demographic information about families headed by gay, lesbian, and heterosexual parents.

			Gay fathers (*n* = 76)	Lesbian mothers (*n* = 122)	Heterosexual parents (*n* = 82)	ANOVA or χ^2^
**Parents (*n* = 263)**						
	Mean age		37.61 (5.61)	33.21 (3.94)	34.85 (4.89)	*F*(2,255) = 17.51, *p* < 0.001
	Ethnic identity, White		89.3%	95.9%	95.5%	χ^2^ (10) = 11.91, *p* = 0.291
	Length of relationship (in years)		6.42 (3.94)	6.75 (2.72)	8.56 (3.52)	*F*(2,260) = 15.41, *p* < 0.001
	Twins, yes		35.5%	5.9%	12.2%	χ^2^ (2) = 28.94, *p* < 0.001, G > L
	Working status, fulltime					χ^2^ (4) = 18.71, *p* = 0.001
		Fulltime	62.9%	55.5%	72.0%	
		Part-time	24.2%	37.0%	11.0%	L > H
		Not working outside home	12.9%	7.6%	17.1%	
	Family Income					χ^2^ (4) = 12.26, *p* = 0.016
		Under 12.706 dollar	0.0%	1.7%	2.4%	
		12.706 – 42.356 dollar	12.9%	35.0%	31.7%	
		Over 42.356 dollar	87.1%	63.2%	65.9%	
	Residency					χ^2^(6) = 24.08, *p* = 0.001
		Rural area	3.2%	4.2%	12.2%	
		Small city	16.1%	37.0%	41.5%	
		Medium city	35.5%	34.5%	26.8%	
		Large city	45.2%	24.4%	19.5%	
**Children *(n* = 146)**						
	Mean age (in months)		3.28 (0.59)	3.42 (0.59)	3.24 (0.64)	*F*(2,143) = 1.27, *p* = 0.283
	Gender (% girls)		57.1%	56.5%	54.3%	χ^2^(2) = 0.08, *p* = 0.962

*Standard deviations are given in parentheses. ANOVA, analysis of variance.*

Additional analyses were performed to identify the source of the significant differences. The family wise Type 1 error rate due to multiple testing was controlled for by using a Bonferroni-corrected α = 0.05/30 = 0.001 as the criterion for statistical significance. Tukey HSD *post hoc* tests showed that gay fathers were significantly older than lesbian mothers (*p* < 0.001). Additional 2 × 2 chi-square analyses showed that gay fathers had twins more often than lesbian mothers did [χ^2^(1) = 21.64, *p* < 0.001]. Lesbian mothers more often worked part-time than heterosexual parents did [χ^2^(1) = 16.05, *p* < 0.001]. Other differences were not statistically significant.

### Procedure

Parents were recruited via specialist lawyers with expertise in surrogacy (for the recruitment of gay fathers), lesbian and gay parenting support groups, fertility clinics (for the recruitment of lesbian and heterosexual parents), and online forums and magazines for gay and lesbian people after ethical approval was granted by the appropriate committees at the three home institutes. Gay fathers were eligible when they had used surrogate carriers, lesbian mothers when they had used sperm donors, and heterosexual parents when they had used IVF without sperm or egg donation to conceive. Only two-parent families with children younger than 4 months and that provided active consent were permitted to participate.

Data were collected twice: when infants were between 3.5 and 4.5 months old (T1) and when they were around 12 months old (T2). The first assessment took place at home and the second assessments at the participating universities. Before the home visits at T1, all parents were queried about their demographic characteristics (including gender and work status) and their infants’ temperament via a unique password protected website. During the home visits, both parents separately completed a password-protected online questionnaire on division of labor (i.e., childcare, household tasks, and family decision making), individual parent characteristics (i.e., depression, anxiety, and parental stress), and partner relationship quality. Before parents came to our institutions for T2, they were again queried about the division of labor using a password-protected online questionnaire. During both visits, other data outside the scope of the current study were also collected (e.g., see Van Rijn - van Gelderen et al. (2018) for further information). The retention rate at T2 for the current analytic sample was 90.9%. In nine families (one Dutch, seven United Kingdom, and one French), both parents did not participate at T2 and in six families one partner dropped out (three Dutch parents, one British parent, and one French parent). Reasons for not participating at T2 included being too busy with a new baby on the way and excessive emotional burden.

### Measures

#### Division of Labor

At both assessments, parents completed the “Who Does What” questionnaire (Cowan and Cowan, 1990) to report on their current experiences with the division of labor within their family. The questionnaire consists of 36 items equally divided over three subscales: household and family tasks (including planning and preparing meals, house cleaning, laundry, looking after the car), family decisions (including plans for social activities and vacations and deciding about the expected behavior of family members), and childcare tasks (including feeding, changing, playing, and doing the baby’s laundry). Each parent was asked to show on a nine-point scale (1 = *I do it all*, 9 = *my partner does everything*) how these tasks were divided between the parents. All scores per subscale were averaged to calculate one score per scale. Internal consistency for the household and family task sub-scale was low at T1 (Cronbach’s α = 0.33) and T2 (Cronbach’s α = 0.31) and thus we decided to focus on childcare tasks and family decisions only. Internal consistency was adequate for the family decisions (Cronbach’s α = 0.65) and high for the childcare tasks (Cronbach’s α = 0.87) sub-scales at 4 months. At 12 months, internal consistency was adequate for family decisions (Cronbach’s α = 0.60) and high for childcare tasks (Cronbach’s α = 0.82).

#### Child Temperament

The fussiness/difficulty subscale (nine items) of the Infant Characteristics Questionnaire (ICQ; Bates et al., 1979) was used to obtain information about the temperament of the infants. Parents rated the fussiness of their infant on a seven-point scale with a low score meaning easy and a high score meaning difficult. An example of the items is: “How easy or difficult is it for you to calm or soothe your baby when he/she is upset?” (1 = *very easy*, 7 = *difficult*). Again, scores were averaged. Internal consistency was high (Cronbach’s α = 0.82).

#### Individual Parent Factors

Parents were asked whether they were biologically related to their infants (0 = *no*, 1 = *yes*) and whether they worked outside the home (0 = *no, not at this moment*, 1 = *yes*). Those who did work outside the home were asked how much they worked (1 = *part-time*, 2 = *fulltime*). The two work-related questions were combined to create one scale to measure work status (0 = *not working outside the home*, 1 = *part-time*, 2 = *fulltime*). Dummy variables were created for not-working (0 = *no*, 1 = *yes*) and part-time (0 = *no*, 1 = *yes*). In addition, standardized questionnaires were used to measure parental psychological adjustment (parental depression, parental anxiety, and parental stress).

##### Parental depression

The Edinburgh Postnatal Depression Inventory (EPDS; Cox et al., 1987) was used to measure depressive symptoms in parents. Parents answered 10 items about their depressive feelings in the past seven days (e.g., “I have been so unhappy that I have been crying” with response categories ranging from 0 = *yes, very often* to 4 = *no, never*). After reversing scores on items reflecting a lack of depression, scores were summed (possible score range: 0 – 30 with scores > 10 indicating a possible major or minor depressive disorder; Cox et al., 1987). Internal consistency was adequate (Cronbach’s α = 0.62).

##### Parental anxiety

Parents’ general level of anxiety was assessed using the Trait Anxiety Scale of the State-Trait Anxiety Inventory – adult version (STAI; Spielberger and Gorsuch, 1983). This scale consists of 20 feelings or emotions and parents rated the frequency of these items on a four-point scale (answer categories ranged from 1 = *almost never* to 4 = *almost always*). An example item is: “I feel nervous and restless.” All item scores were summed after responses to items reflecting an absence of anxiety (e.g., “I am happy”) were reversed. Scores ranged from 20 to 80, with higher scores reflecting a higher level of anxiety (scores > 44 indicate high anxiety). Internal consistency was high (Cronbach’s α = 0.86).

##### Parental stress

Parents completed the subscale Parental Distress of the short version of the Parenting Stress Index (PSI; Abidin, 2012) to report on their levels of parental stress. An example of the 12 items in this subscale is “I feel trapped by my responsibilities as a parent” with response categories ranging from 1 (*strongly agree*) to 5 (*strongly disagree*). Scores were summed to create a total score. Scores > 33 indicates high parental stress (Abidin, 2012). Internal consistency was high (Cronbach’s α = 0.85).

#### Partner Relationship Quality

Partner relationship quality was assessed using the Golombok Rust Inventory of Marital State (GRIMS; Rust et al., 1986). This questionnaire consists of 28 items (e.g., “I am dissatisfied with our relationship”) and parents had to rate these items on a scale of 0 (*strongly agree*) to 3 (*strongly disagree*). Half of the items are positively formulated and the other half are negatively formulated. In accordance with the GRIMS manual, the sum of negative items was subtracted from the sum of positive items, and then 42 was added to create the raw GRIMS score. Higher scores indicate poorer relationship quality (scores > 42 indicate severe relationship problems) (Rust et al., 1986).

### Statistical Analyses

Our first aim was to determine which factors (child temperament, individual parent characteristics, and partner relationship quality) were related to family decisions making and childcare tasks at 4 months and at 12 months. To do so, we performed four linear mixed models with child temperament, individual parent characteristics (i.e., biological relatedness to child, work status, psychological adjustment, parenting stress), and partner relationship quality as parameters. Our second aim was to see whether significant parameters were the same for each family type. We used Hayes’ PROCESS module for SPSS (Hayes, 2017) to test whether the relation between significant parameters and the corresponding outcome variables were moderated by family type.

In the literature, missing values are often deleted in a pair-wise way. However, such methods lead to the introduction of (unwanted) bias and reduce power (Enders, 2010; Graham, 2012). Modern treatments for missing data, such as multiple imputation, provide effective solutions to these problems (Little et al., 2014) and can be used for dichotomous data too (Wu et al., 2015). To minimize bias and optimize power, missing data in this study (both T2 drop-outs and single missing items; see note on Table 2 for specific numbers) were therefore handled by multiple imputation. Analysis of multiply imputed data involves three steps. First, we estimated missing values *m* times, resulting in *m* plausible complete versions of the incomplete data set. We used *m* = 20 imputations, using the “fully conditional specification” available in IBM SPSS 25.0 (2017). Second, each imputed data set was analyzed using the same statistical analysis applicable for complete data. Third, the results from each of the *m* = 20 analyses were combined into a single set of “pooled” results, using Rubin’s (1987) rules for pooling estimates and *SEs* across imputations. We used the SPSS macro provided by Van Ginkel (2010) to perform the analysis and pooling steps in IBM SPSS 25.0 (2017), which estimates the (denominator) degrees of freedom for *t* (or *F*) statistics using the robust method described by Van Ginkel and Kroonenberg (2014, p. 80, eq. 7). When there were significant interactions, we probed the interaction by applying Hayes’ (2017) PROCESS macro for SPSS to each imputed data set, and then pooled moderation results using Rubin’s rules.

**TABLE 2:** Means and standard errors of division of labor, infant temperament, individual parent factors, and partner relationship quality by family type.

	Gay fathers (*n* = 62)	Lesbian mothers (*n* = 119)	Heterosexual parents (*n* = 82)	Females (*n* = 160)	Males (*n* = 103)	Parent A (*n* = 133)	Parent B (*n* = 130)	Total (*n* = 263)
*At four months*								
**Division of labor**								
Family Decisions^*a*^	4.91 (0.09)	4.99 (0.05)	5.07 (0.07)	5.00 (0.05)	5.00 (0.07)	4.95 (0.06)	5.05 (0.05)	5.00 (0.04)
Child Care Tasks^*b,j,k,l*^	4.88 (0.10)	4.92 (0.08)	4.94 (0.18)	4.61 (0.08)	5.41 (0.10)	4.20 (0.08)	5.66 (0.07)	4.92 (0.07)
Infant Temperament^*c*^	2.74 (0.10)	3.03 (0.07)	2.98 (0.08)	3.04 (0.06)	2.79 (0.07)	3.00 (0.06)	2.89 (0.07)	2.95 (0.05)
**Individual Parent Factors**								
Biological relatedness, yes	50%	52.9%	100%	65%	69.9%	89.5%	43.8%	66.9%
Parental Depression^*d*^	3.87 (0.34)	4.54 (0.27)	4.58 (0.32)	4.72 (0.23)	3.88 (0.26)	4.65 (0.26)	4.13 (0.24)	4.39 (0.18)
Parental Anxiety^*e*^	31.39 (0.87)	33.69 (0.66)	33.33 (0.85)	33.75 (0.59)	31.91 (0.69)	33.34 (0.68)	32.72 (0.59)	33.03 (0.45)
Parental Stress^*f*^	21.63 (1.15)	21.55 (0.57)	22.31 (0.58)	21.81 (0.47)	21.81 (0.78)	22.60 (0.60)	20.99 (0.57)	21.81 (0.41)
Partner relationship quality^*g*^	21.14 (1.27)	20.14 (0.75)	21.86 (0.84)	20.40 (0.64)	21.71 (0.89)	21.10 (0.75)	20.73 (0.72)	20.91 (0.52)
*At 12 months*								
**Division of labor**								
Family Decisions^*h*^	4.99 (0.08)	4.95 (0.05)	5.10 (0.08)	4.97 (0.05)	5.07 (0.06)	4.95 (0.05)	5.06 (0.05)	5.01 (0.04)
Child Care Tasks^*i,j,k,l*^	4.96 (0.10)	4.93 (0.08)	4.91 (0.14)	4.72 (0.08)	5.25 (0.09)	4.51 (0.08)	5.36 (0.07)	4.93 (0.06)

*Calculated from dataset with imputations (pooled). Numbers of missing values in at least one of the questions in the questionnaire: ^*a*^*n* = 22 (8.4%), ^*b*^*n* = 8 (3.0%), ^*c*^*n* = 9 (3.4%), ^*d*^*n* = 4 (1.5%), ^*e*^*n* = 15 (5.7%), ^*f*^*n* = 4 (1.5%), ^*g*^in first part of questionnaire: *n* = 8 (3.5%) and in second part of the questionnaire: *n* = 4 (1.5%), ^*h*^*n* = 44 (16.7%), ^*i*^*n* = 27 (10.3%), ^*j*^Heterosexual mothers spent on average more time on childcare tasks at 4 months and 12 months than lesbian mothers did, *p* < 0.001 on both waves, ^*k*^Gay fathers spent on average more time on childcare tasks at 4 months and 12 months than heterosexual fathers did, *p* < 0.001 on both waves. ^*l*^Mothers spent on average more time on childcare tasks at 4 and 12 months than fathers did, *p* < 0.001 on both waves.*

To distinguish between caregivers, we labeled them caregiver A and caregiver B. The answer to the question “During the past week, who spent the most time with [name infant(s)]?” (asked by the research assistant when arranging the home visit) was used to identify caregiver A. The other parent was automatically identified as caregiver B. Caregiver A and caregiver B were randomly assigned when parents stated that they spend equal time with the infant(s). In addition, we randomly selected one twin for each family with twins, to avoid using the same parental scores twice.

## Results

### Preliminary Analyses

Descriptive statistics for the total group, as well as for the statistics by gender, family type, and by caregiver (A or B) are presented in Table 2. To give an overview of the amount of imputed data, this table also shows the number of incomplete cases per questionnaire for the total group. Correlations between variables are presented in Table 3. Prior to the hierarchical regression analyses, the assumptions for this test were checked^1^.

**TABLE 3:** Correlations between division of labor, infant temperament, individual parent factors, and interpersonal factors.

	1	2	3	4	5	6	7	8	9
(1) Family Decisions at 4 months	1								
(2) Childcare Tasks at 4 months	0.24**	1							
(3) Infant Temperament	0.05	–0.04	1						
(4) Parental Depression	–0.01	–0.07	0.15*	1					
(5) Parental Anxiety	0.06	–0.03	0.26**	0.65**	1				
(6) Parental Stress	0.02	–0.06	0.23**	0.36**	0.39**	1			
(7) Partner relationship quality	–0.06	0.01	0.06	0.31**	0.41**	0.33**	1		
(8) Family Decisions at 12 months	0.50**	0.25**	0.05	0.02	0.11	0.09	0.01	1	
(9) Childcare Tasks at 12 months	0.10	0.62**	0.02	–0.01	0.00	–0.05	0.04	0.24**	1

Calculated from dataset with imputations (pooled). *p < 0.05, **p < 0.001.

The data for family decisions at T1 and T2, anxiety, parenting stress scores at T1 were slightly peaked and slightly skewed. These deviations from the normal distribution seemed to be caused by some outliers^2^. Sensitivity analyses were conducted to see whether the results differed when outliers were excluded and the results were similar.

### Parameters of Task Division at Four Months

#### Family Decision Making

No significant equation was found [*F*(8,260.99) = 0.819, *p* = 0.586] when we assessed a mixed linear model with family decision making as the dependent variable and with infant temperament, biological relatedness, parental depression, parental anxiety, parenting stress, work status (not working vs. fulltime), work status (part-time vs. fulltime), and parent relationship quality as parameters.

#### Childcare Tasks

The mixed linear model with childcare tasks as the dependent variable and with infant temperament, biological relatedness, parental depression, parental anxiety, parenting stress, work status (not working), work status (part-time), and parent relationship quality as parameters showed that *R*^2^ was significantly different from zero, *F*(8,261.02) = 4.64, *p* < 0.001. Results showed that biological relatedness and work status (both not working vs. fulltime and part-time vs. fulltime) significantly contributed to the equation (see Table 4 for the estimates of the fixed effects of the parameters in the model). Parents who were biological related to their children scored lower on relative time spend on childcare tasks, indicating that they were doing more of the childcare tasks than their partners were doing. Results with regard to work status showed similar results: compared to those who worked full-time, parents working less (either not working outside the home or working part-time) reported lower scores on the childcare task sub-scale (indicating that they were doing more than their partner).

**TABLE 4:** Fixed effects estimates of predictors of childcare tasks.

	Childcare Division at 4 months			Childcare Division at 12 months		
Parameter	Estimate (*SE*)	95% Confidence Interval		Estimate (SE)	95% Confidence Interval	
		*LL*	*UL*		*LL*	*UL*
Intercept	5.58(0.41)**	4.79	6.38	5.13(0.35)**	4.63	6.00
Infant temperament	−0.02(0.09)	–0.20	0.16	−0.00(0.08)	–0.16	0.16
**Individual parent characteristics**						
Biological relatedness	−0.41(0.14)*	–0.69	–0.14	−0.15(0.12)	–0.39	0.09
Parental Depression	−0.03(0.03)	–0.09	0.03	−0.01(0.03)	–0.06	0.05
Parental Anxiety	0.01 (0.01)	–0.02	0.03	0.01 (0.01)	–0.02	0.03
Parenting stress at 4 months	−0.01(0.01)	–0.03	0.01	−0.01(0.01)	–0.03	0.01
Work status at 4 months – not working vs. fulltime	−0.95(0.21)**	–1.36	–0.54	−0.60(0.20)**	–1.00	–0.21
Work status at 4 months- Part-time vs. fulltime	−0.44(0.16)*	–0.75	–0.14	−0.05(0.17)	–0.38	0.29
Work status at 12 months – not working vs. fulltime	n/a			−0.78(0.22)**	–1.22	–0.34
Work status at 12 months- Part-time vs. fulltime	n/a			−0.38(0.16)*	–0.70	–0.06
Partner relationship quality	−0.00(0.01)	–0.02	0.02	0.00 (0.01)	–0.01	0.02

Calculated from dataset with imputations (pooled). *p < 0.05, **p < 0.001.

### Parameters of Task Division at 12 Months

#### Family Decision Making

The same analysis was conducted with family decision making at 12 months as the dependent variable but also with the two work status variables at 12 months included. Again, no significant equation was found, *F*(10,259.08) = 0.82, *p* = 0.609.

#### Childcare Tasks

For childcare tasks at 12 months, *R*^2^ was significantly different from zero, *F*(10,260.08) = 4.63, *p* < 0.001. The mixed linear model with childcare tasks at 12 months as the dependent variable showed that the division of childcare tasks at this time point was only related to work status at 4 months (not working vs. working fulltime) and work status at 12 months (not working vs. working fulltime and part-time working vs. working fulltime); see Table 4 for the estimates of the fixed effects of the parameters in the model. Not working when the baby was 4 months old was related to spending more time on childcare tasks at 12 months than were parents who were working fulltime at 4 months. Not working and working part-time when the baby was 12 months old was related to spending more time on childcare tasks than parents who were working fulltime at 12 months.

### Moderation Analyses

First, we checked whether the relation between biological relatedness and childcare tasks at 4 months was moderated by family type. We excluded heterosexual parents, because they were all biologically related to their children (see Table 2). Results showed that, for lesbian mothers, biological relatedness was related to spending more time on childcare tasks than their partners *(pooled estimate* = *−*0.95, *pooled SE* = 0.49; 95% CI: *LL* = −1.99, *UL* = 0.08). For gay fathers, the relation between childcare task involvement and biological relatedness was not significant (*pooled estimate* = 0.26, *pooled SE* = 0.19; 95% CI: *LL* = −0.13, *UL* = 0.65). Second, we analyzed whether family type also acted as a moderator for the relation between (a) not working vs. fulltime and (b) working part-time vs. fulltime and time spent on childcare tasks at 4 months. The moderation results revealed that family type was not a significant moderator. Model results ranged from *R*^2^ (2,257) = 0.01, *p* = 0.457 to *R*^2^ (2,257) = 0.01, *p* = 0.442 for not working vs. full-time. For part-time vs. fulltime working, the model results ranged from *R*^2^ (2,257) = 0.01, *p* = 0.244 to *R*^2^ (2,257) = 0.11, *p* = 0.236. Likewise, family type did not act as a moderator for associations between the three parameters (not working vs. fulltime at 4 months, not working vs. fulltime at 12 months, and part-time vs. fulltime at 12 months) and time spent on childcare tasks at 12 months. Model results ranged from *R*^2^ (2,257) = 0.01, *p* = 0.473 to *R*^2^ (2,257) = 0.01, *p* = 0.120 for not working vs. full-time at 4 months. For not working vs. fulltime at 12 months the model results ranged from *R*^2^ (2,257) = 0.00, *p* = 0.746 to *R*^2^ (2,257) = 0.01, *p* = 0.202 and for part-time working vs. fulltime at 12 months from *R*^2^ (2,257) = 0.00, *p* = 0.663 to *R*^2^ (2,257) = 0.01, *p* = 0.265. Thus, for all parents, irrespective of family type, work status was related to relative time spend on childcare tasks at 4 months and at 12 months in the same way (parents working less reported spending more time on childcare tasks).

## Discussion

In identifying the determinants of the division of non-paid tasks between parents, we drew from Feinberg’s (2003) model of co-parenting. We investigated whether child temperament, individual parent characteristics (i.e., biological relatedness to child, work status, psychological adjustment, parenting stress), and partner relationship quality explained how first-time gay, lesbian, and heterosexual parents divided labor (family decision making and childcare tasks) when their infants were 4 and 12 months old. Results showed that none of the factors explained the division of family decision making at 4 and 12 months. For relative time spent on childcare tasks, we found that biological relatedness mattered: parents who were biologically related to their children appeared to spend more time on childcare tasks than did non-related parents. However, this was only true for the lesbian mothers, and, interestingly, only when their children were 4 months old. In addition, parents who were not working or were working part-time at 4 months performed more childcare tasks at 4 months while not working and working part-time when the baby was 12 months old was also related to spending more time on childcare tasks at 12 months relative to parents who were working fulltime at 12 months. This was true for all family types. Other factors were not related to the relative amounts of time parents spent on childcare tasks.

All heterosexual parents were biologically related to their children, so we were unable to investigate whether variance within this group was explained by biological relatedness. For gay fathers, biological relatedness did not predict relative involvement in childcare tasks. This is not in line with the theory of selection (Hamilton, 1964), which suggests that biologically related parents invest more in their children than non-biological parents do. A plausible explanation is that gay fathers have a very unique position in our society. It is still rare for men to be primary caregivers and it is commonly supposed that men are less nurturing (Golombok et al., 2014). Artificial reproductive techniques that were used by lesbian mothers and heterosexual parents in our study are much more available in current society, while surrogacy is not. For example, in the Netherlands at the time of the study, gestational surrogacy was only available for medical reasons –excluding gay couples (Boele-Woelki et al., 2011). Gay fathers therefore have to overcome more obstacles before they are able to conceive (Taubman-Ben-Ari and Spielman, 2014) which could make them highly motivated to take care of their children – irrespective of whether they are biologically related or not. Finally, a substantial number of gay fathers with twins were biologically related to one of the twins. We only selected one twin for each family, and thus some gay fathers treated as non-biological fathers in the analyses were biologically related to the other infant in the family. This might have increased the amount of time spent on childcare tasks. More research is needed to see whether this idea is supported by data.

The results for lesbian mothers were slightly different; they were partially in line with both social structural theory (Eagly and Wood, 1999), which assumes that biological abilities are related to the roles people play, and earlier studies of lesbian mothers with infants (e.g., Goldberg et al., 2012). At 4 months, biological mothers were spending more time on childcare tasks than non-biologically related mothers. This sounds plausible because birth mothers usually have greater access to paid parental leave (Goldberg, 2010) and are more likely to breastfeed. After 12 months, the link between biological relatedness and relative investment in childcare tasks disappeared. This supports our notion that the relation between biological status and time spend on childcare at 4 months it not driven by biological status itself but more by factors related to giving birth. Another explanation might be that non-biological parents, because of the lack of a biological link to the infants, are more motivated to spend time caring for the infants when they are older, perhaps feeling that more work is needed to establish meaningful relationships with the children. The biologically related parents, on the other hand, may be particularly sensitive to the partners’ position and may attempt to support the relationships between children and the non-biological related parents (Johnson and O’Connor, 2002), resulting in a more equitable division of childcare tasks. Future research should investigate this in more depth.

As hypothesized, childcare task division at 4 and 12 months was related to how much parents worked regardless of family type: those who worked less than full-time, spent more time on childcare tasks. This is in line with both the time-constraint theory (Artis and Pavalko, 2003), which states that there are only an finite number of hours during the day and that those who spend more time at paid work have less time available for non-paid work, and empirical studies of same-sex and different-sex parent families (Downing and Goldberg, 2011; Goldberg et al., 2012; Tornello et al., 2015). It is not in line with the results of a study on adoptive gay fathers (Feuge et al., 2019). However, that study focused on a broader topic, namely parental involvement, measured using questions about emotional support, discipline, physical care, openness to work, physical play, and evocation (thinking about the child in his/her absence) (Feuge et al., 2019). Parental involvement thus included activities that can be performed while working outside the home, such as thinking about the child. This might explain the absence of a link between work hours and parental involvement in that study.

Interestingly, we did not find any evidence that Feinberg’s (2003) model of co-parenting is also applicable to the division of family decision making, suggesting that the decision-making process is influenced by other factors. One explanatory factor might be the amount of time spent on household tasks. For example, Moore (2008) found that Black lesbian-headed stepmothers who were in charge of domestic duties also reported that they were more in charge of major household decisions. Bartley et al. (2005) studied family decision-making in a group of heterosexual dual-earner couples without children and found similar results. Wives tended to spend more time on household tasks and tended to perceive themselves as more influential in decision making than their husbands. Unfortunately, our measure of relative time spent on household tasks was not reliable so we could not test this idea. Future studies on gay fathers and lesbian mothers with infants could test whether family decision making in same-sex parent families with young infants is also related to time spent on household tasks.

Gender still affects the division of non-paid tasks. Females spend more time on non-paid tasks than males do (Baxter et al., 2008, 2015), presumably because of gendered roles and gender ideology on a societal level (Geist, 2005; Greenstein, 2009; Nyman et al., 2013). Although it was beyond the scope of our research, it would be interesting to know how lesbian mothers and gay fathers identify with such traditional and/or non-traditional gender roles or expressions. In addition, future qualitative research might address questions like: do gay fathers feel equally involved in parenting? Why do lesbian mothers perform more part-time jobs than heterosexual mothers?

This study was the first to provide information about non-paid task division by gay fathers, lesbian mothers, and heterosexual parents with infants. Also, it was the first to use a more general model (Feinberg’s model of co-parenting) to investigate possible determinants of non-paid task division, although most factors in the model were not influential; only work status was related to relative time spent on childcare tasks at 12 months. Further, because we used data from two waves (4 months and at 12 months), it was possible to detect any changes in determinants across the first year of parenthood. Furthermore, we had information from both parents for most families (*n* = 133).

Of course, the study also had some limitations. Unfortunately, we did not have reliable data about the relative amounts of time spent on household tasks. A reason for the low internal consistency of our measure might be the mix of stereotypically feminine, masculine, and neutral tasks included in the household tasks scale (Sumontha et al., 2017). Another limitation concerning the sample is the relatively high socioeconomic status and White ethnic background. This limits the generalizability to the whole population of first-time parents from heterosexual, gay–father, and lesbian–mother families although it is noteworthy that most gay fathers who used surrogacy to conceive are similar to those we studied. Surrogacy is very costly (between $90,000 and more than $120,000 in the US) (Thompson and Dodge, 2018) and therefore only an option for couples with high incomes. The non-probability techniques that were used to recruit the families also hamper generalizability (Bryman, 2012). Unfortunately, due to the sample size we were not able to analyze data for the parents in the Netherlands, France, and the United Kingdom separately. In the future, larger studies should explore this because parental leave policies vary greatly internationally. For example, Dutch mothers can take up 10 weeks of maternity leave^3^, French mothers 20 weeks, and British mothers around 50 weeks (Van Belle, 2016) albeit with very different levels of income. Finally, it would have been interesting to have a comparison group of couples who naturally conceived to see whether the findings of the current study would be the same or are specific to families who had to use artificial reproductive techniques to conceive.

Notwithstanding these limitations, our study gave us the opportunity to examine the division of non-paid tasks in families where parenting is always planned, as well as in families wherein gender is not a factor in that division of labor. Although Feinberg’s model of co-parenting suggests that various factors other than gender are related to task division, our results showed that paid work outside the home was of great importance. Indeed, work hours at 4 and 12 months were the only significant correlates of relative time spent at 12 months. Our findings might encourage counselors who guide gay, lesbian, or heterosexual parents who are candidates for artificial reproductive techniques by talking to prospective parents about the link between paid and non-paid tasks to help them decide how to divide roles in their future families. Also, to decrease the still existing gender gap, with women spending more time on childcare tasks than men (Baxter et al., 2015), governments might also give secondary caregivers the option to decrease their work hours at 4 months so that childcare tasks might be divided more equitably at 12 months.

## Data Availability Statement

The datasets generated for this study are available on request to the corresponding author.

## Ethics Statement

Ethical approval was granted by the appropriate committees at the three home institutes, namely University of Cambridge, University of Amsterdam, and Centre Universitaire des Saints-Pères. Written informed consent to participate in this study was provided by the participants’ legal guardian/next of kin.

## Author Contributions

ML, HB, and OV were responsible for the design of the study in collaboration with MG, KE-D, AW, LV, and BR. LV, HB, KE-D, AW, BR, OV, and MG collected the data. KD was overseen the management of the study. TJ and LV conducted the data analysis. LV and MH-E interpreted the results and drafted this manuscript. All authors contributed to the final version and have approved the final version for submission.

## Conflict of Interest

The authors declare that the research was conducted in the absence of any commercial or financial relationships that could be construed as a potential conflict of interest.

## Footnotes

1 Since the data were nested, we checked whether it was necessary to account for the dependency of the data. Null models with random effects for infants, parents, and couples indicated that no variance was explained by any of these levels.

2 We found extreme scores on family decision making at T1 (two per dataset), anxiety (one per dataset), distress (two per dataset), not working (four per dataset), and family decision making at T2 (2 in the original dataset and 1 per imputed dataset) to be univariate outliers. Two cases in the original dataset and three cases in the imputation sets (including two from the same family) were identified through Mahalanobis distance as multivariate outliers with *p*s < 0.001.

3 www.rijksoverheid.nl

## References

[B1] Abidin R. R. (2012). *Parenting Stress Index.* Lutz, FL: PAR.

[B2] Artis J. E. Pavalko E. K. (2003). Explaining the decline in women’s household labor: Individual change and cohort differences. *J. Marriage Fam.* 65 746–761.

[B3] Bartley S. J. Blanton P. W. Gilliard J. L. (2005). Husbands and wives in dual-earner marriages: decision-making, gender role attitudes, division of household labor, and equity. *Marriage Fam. Rev.* 37 69–94. 10.1300/J002v37n04_05

[B4] Bates J. E. Bennett Freeland C. A. Lounsbury M. L. (1979). Measurement of infant difficultness. *Child Dev.* 50 794–803. 10.2307/1128946 498854

[B5] Baxter J. Buchler S. Perales F. Western M. (2015). A life-changing event: first births and men’s and women’s attitudes to mothering and gender divisions of labor. *Soc. Forc.* 93 989–1014. 10.1093/sf/sou103

[B6] Baxter J. Hewitt B. Haynes M. (2008). Life course transitions and housework: marriage, parenthood, and time on housework. *J. Marriage Fam.* 70 259–272.

[B7] Bianchi S. M. Sayer L. C. Milkie M. A. Robinson J. P. (2012). Housework: who did, does or will do it, and how much does it matter? *Soc. Forc.* 91 55–63. 10.1093/sf/sos120 25429165 PMC4242525

[B8] Boele-Woelki K. Curry-Sumner I. Schrama W. Vonk M. (2011). *Draagmoederschap En Illegale Opneming Van Kinderen [Commercial Surrogacy And The Unlawful Placement Of Children].* The Hague: WODC.

[B9] Bos H. M. W. Van Balen F. Van den Boom D. C. (2007). Child adjustment and parenting in planned lesbian-parent families. *Am. J. Orthopsychiatry* 77 38–48. 10.1037/0002-9432.77.1.38 17352583

[B10] Bryman A. (2012). *Social Research Methods*, 4th Edn, Oxford: Oxford University Press.

[B11] Burney R. V. Leerkes E. M. (2010). Links between mothers’ and fathers’ perceptions of infant temperament and coparenting. *Infant Behav. Dev.* 33 125–135. 10.1016/j.infbeh.2009.12.002 20116859 PMC2844911

[B12] Cao H. Milles-Koonce W. R. Wood C. Fine M. A. (2016). Identity transformation during the transition to parenthood among Same-Sex couples: an ecological, stress-strategy-adaptation perspective. *J. Fam. Theor. Rev.* 8 30–59. 10.1111/jftr.12124 27458482 PMC4957560

[B13] Chan R. W. Raboy B. Patterson C. J. (1998). Psychosocial adjustment among children conceived via donor insemination by lesbian and heterosexual mothers. *Child Dev.* 69 443–457. 9586218

[B14] Claffey S. T. Mickelson K. D. (2009). Division of household labor and distress: the role of perceived fairness for employed mothers. *Sex Roles* 60 819–831. 10.1007/s11199-008-9578-0

[B15] Coltrane S. (2000). Research on household labor: modeling and measuring the social embeddedness of routine family work. *J. Marriage Fam.* 62 1208–1233.

[B16] Cooper C. E. McLanahan S. S. Meadows S. O. Brooks-Gunn J. (2009). Family structure transitions and maternal parenting stress. *J. Marriage Fam.* 71 558–574. 10.1111/j.1741-3737.2009.00619.x 20046951 PMC2743274

[B17] Cowan C. P. Cowan P. A. (1990). “Who does what?,” in *Handbook of Family Measurement Techniques*, eds Touliatos J. Perlmutter B. F. Straus M. A. (Beverly Hills, CA: Sage), 447–448.

[B18] Cowan C. P. Cowan P. H. (1988). Who does what when partners become parents: Implications for men, women and marriage. *Marriage Fam. Rev.* 12 105–131. 10.1300/J002v12n03_07

[B19] Cox J. L. Holden J. M. Sagovsky R. (1987). Detection of postnatal depression: development of the 10-item edinburgh postnatal depression scale. *Br. J. Psychiatry* 150 782–786. 10.1192/bjp.150.6.782 3651732

[B20] Downing J. B. Goldberg A. E. (2011). Lesbian mothers’ constructions of the division of paid and unpaid labor. *Femin. Psychol.* 21 100–120. 10.1177/0959353510375869

[B21] Durtschi J. A. Soloski K. L. Kimmes J. (2017). The dyadic effects of supportive coparenting and parental stress on relationship quality across the transition to parenthood. *J. Marit. Fam. Therapy* 43 308–321. 10.1111/jmft.12194 27701778

[B22] Eagly A. H. Wood W. (1999). The origins of sex differences in human behavior: evolved dispositions versus social roles. *Am. Psychol.* 54 408–423. 10.1037/0003-066X.54.6.408

[B23] Earle S. (2000). Why some women do not breast feed: bottle feeding and fathers’ role. *Midwifery* 16 323–330. 10.1054/midw.2000.0222 11080468

[B24] Ehrenberg M. F. Gearing-Small M. Hunter M. A. Small B. J. (2001). Childcare task division and shared parenting attitudes in dual-earner families with young children. *Fam. Relat.* 50 143–153. 10.1111/j.1741-3729.2001.00143.x

[B25] Enders C. K. (2010). *Applied Missing Data Analysis.* New York, NY: Guilford.

[B26] Farr R. H. Patterson C. J. (2013). Coparenting among lesbian, gay, and heterosexual couples: Associations with adopted children’s outcomes. *Child Dev.* 84 1226–1240. 10.1111/cdev.12046 23336749

[B27] Feinberg M. E. (2003). The internal structure and ecological context of coparenting: a framework for research and intervention. *Parent. Sci. Pract.* 3 95–131. 10.1207/S15327922PAR0302_01 21980259 PMC3185375

[B28] Feuge E. A. Cossette L. Cyr C. Julien D. (2019). Parental involvement among adoptive gay fathers: associations with resources, time constraints, gender role, and child adjustment. *Psychol. Sex. Orient. Gender Divers.* 6 1–10. 10.1037/sgd0000299

[B29] Gamble D. Morse J. M. (1993). Fathers of breastfed infants: postponing and types of involvement. *J. Obstet. Gynecol. Neonat. Nurs.* 33 358–365. 10.111/j.1552-6909.1993.tb01816.x 8410435

[B30] Gartrell N. Banks A. Hamilton J. Reed N. Bishop H. Rodas C. (1999). The national lesbian family study: 2. interviews with mothers of toddlers. *Am. J. Orthopsychiatry* 69 362–369. 10.1037/h0080410 10439850

[B31] Gartrell N. Banks A. Reed N. Hamilton J. Rodas C. Deck A. (2000). The national lesbian family study: 3. interviews with mothers of five-year-olds. *Am. J. Orthopsychiatry* 70 542–548. 10.1037/h0087823 11086532

[B32] Geist C. (2005). The welfare state and the home: regime differences in the domestic division of labour. *Eur. Sociol. Rev.* 21 23–41.

[B33] Glass J. Fujimoto T. (1994). Housework, paid work, and depression among husbands and wives. *J. Health Soc. Behav.* 35 179–191. 10.2307/2137364 8064124

[B34] Goldberg A. E. (2010). *Lesbian and Gay Parents And Their Children: Research On The Family Life Cycle.* Washington, DC: American Psychological Association.

[B35] Goldberg A. E. Perry-Jenkins M. (2004). Division of labor and working-class women’s well-being across the transition to parenthood. *J. Fam. Psychol.* 18 225–236. 10.1037/0893-3200.18.1.225 14992623 PMC2834188

[B36] Goldberg A. E. Perry-Jenkins M. (2007). The division of labor and perceptions of parental roles: lesbian couples across the transition to parenthood. *J. Soc. Pers. Relationsh.* 24 297–318. 10.1177/0265407507075415

[B37] Goldberg A. E. Smith J. Z. Perry-Jenkins M. (2012). The division of labor in lesbian, gay, and heterosexual new adoptive parents. *J. Marriage Fam.* 74 812–828. 10.1111/j.1741-3737.2012.00992.x

[B38] Golombok S. Mellish L. Jennings S. Casey P. Tasker F. Lamb M. E. (2014). Adoptive gay father families: parent–child relationships and children’s psychological adjustment. *Child Dev.* 85 456–468. 10.1111/cdev.12155 24033323 PMC4510787

[B39] Graham J. W. (2012). *Missing Data: Analysis And Design.* New York, NY: Springer.

[B40] Greenstein T. N. (2009). National context, family satisfaction, and fairness in the division of household labor. *J. Marriage Fam.* 71 1039–1051. 10.1111/j.1741-3737.2009.00651.x

[B41] Gross C. L. Marcussen K. (2017). Postpartum depression in mothers and fathers: the role of parenting efficacy expectations during the transitions to parenthood. *Sex Roles* 76 290–305. 10.1007/s11199-016-0629-7

[B42] Grote N. K. Naylor K. Clark M. S. (2002). Perceiving the division of family work to be unfair: do social comparisons, enjoyment, and competence matter? *J. Fam. Psychol.* 16 510–522. 10.1037//0893-3200.16.4.510 12561295

[B43] Hall L. D. Walker A. J. Acock A. C. (1995). Gender and family work in one-parent households. *J. Marriage Fam.* 57 685–692.

[B44] Hamilton W. D. (1964). The genetical evolution of social behavior. I. *J. Theor. Biol.* 7 1–16. 10.1016/0022-5193(64)90038-4 5875341

[B45] Hayes A. F. (2017). *Introduction To Mediation Moderation And Conditional Process Analysis: A Regression-Based Approach*, 2nd. Edn, New York, NY: Guilford.

[B46] Heron J. O’Connor T. G. Evans J. Golding J. Glover V. The ALSPAC Study Team (2004). The course of anxiety and depression through pregnancy and the postpartum in a community sample. *J. Affect. Disord.* 80 65–73. 10.1016/j.jad.2003.08.004 15094259

[B47] Johnson S. M. O’Connor E. (2002). *The Gay Baby Boom: The Psychology Of Gay Parenthood.* New York, NY: New York University Press.

[B48] Katz-Wise S. L. Priess H. A. Hyde J. S. (2010). Gender-role attitudes and behavior across the transition to parenthood. *Dev. Psychol.* 46 18–28. 10.1037/a0017820 20053003 PMC3764615

[B49] Lennon M. C. Rosenfield S. (1994). Relative fairness and the division of housework: the importance of options. *Am. J. Sociol.* 100 506–531.

[B50] Little T. D. Jorgensen J. D. Lang K. M. Moore E. W. G. (2014). On the joys of missing data. *J. Pediat. Psychol.* 39 151–162. 10.1093/jpepsy/jst048 23836191

[B51] McHale J. P. Kazali C. Rotman T. Talbot J. Carleton M. Lieberson R. (2004). The transition to coparenthood: parents’ prebirth expectations and early coparental adjustment at 3 months postpartum. *Dev. Psychopathol.* 16 711–733. 10.1017/S0954579404004742 15605633

[B52] Minuchin P. (1985). Families and individual development: provocations from the field of family therapy. *Child Dev.* 56 289–302. 10.2307/1129720 3886321

[B53] Moore M. R. (2008). Gendered power relations among women: a study of household decision making in black, lesbian stepfamilies. *Am. Sociol. Rev.* 73 335–356. 10.1177/000312240807300208

[B54] Mulsow M. Caldera Y. M. Pursley M. Reifman A. Huston A. C. (2004). Multilevel factors influencing maternal stress during the first three years. *J. Marriage Fam.* 64 944–956.

[B55] Musick K. Meier A. Flood S. (2016). How parents fare: mothers’ and fathers’ subjective well-being in time with children. *Am. Sociol. Rev.* 81 1069–1095. 10.1177/0003122416663917

[B56] Nyman C. Reinikainen L. Stocks J. (2013). Reflections on a cross-national qualitative study of within-household finances. *J. Marriage Fam.* 75 640–650. 10.1111/jomf.12033

[B57] Ostberg M. (1998). Parental stress, psychosocial problems and responsiveness in help-seeking parents with small (2-45 months old) children. *Acta Psychiatr. Scand.* 87 69–76. 10.1080/08035259850157903 9510451

[B58] Patterson C. J. Sutfin E. L. Fulcher M. (2004). Division of labor among lesbian and heterosexual parenting couples: correlates of specialized versus shared patterns. *J. Adult Dev.* 11 179–189.

[B59] Patterson J. M. (1988). Families experiencing stress: I. the family adjustment and adaptation response model: II. applying the FAAR model to health-related issues for intervention and research. *Fam. Syst. Med.* 6 202–237.

[B60] Rothbart M. K. Bates J. E. (1998). “Temperament,” in *Handbook of Child Psychology: Vol. 3. Social Emotional And Personality Development*, 3rd Edn, eds Eisenberg N. Damon W. (New York, NY: Wiley), 105–176.

[B61] Rubin D. B. (1987). *Multiple Imputation For Nonresponse In Surveys.* New York, NY: John Wiley.

[B62] Rust J. Bennun I. Crowe M. Golombok S. (1986). The golombok rust inventory of marital state (GRIMS). *J. Sex Marit. Therapy* 1 55–60. 10.1080/02674658608407680

[B63] Solmeyer A. R. Feinberg M. E. (2011). Mother and father adjustment during early parenthood: the roles of infant temperament and coparenting relationship quality. *Infant Behav. Dev.* 34 504–514. 10.1016/j.infbeh.2011.07.006 21868100 PMC3172346

[B64] Spielberger C. D. Gorsuch R. L. (1983). *State-Trait Anxiety Inventory For Adults: Manual Instrument And Scoring Guide.* Redwood City, CA: Mind Garden.

[B65] Stone L. L. Mares S. H. W. Otten R. Engels R. C. M. E. Janssens J. M. A. M. (2016). The co-development of parenting stress and childhood internalizing and externalizing problems. *J. Psychopathol. Behav. Assess.* 38 76–86. 10.1007/s10862-015-9500-3 27069304 PMC4789299

[B66] Sumontha J. Farr R. H. Patterson C. J. (2017). Children’s gender development: associations with parental sexual orientation, division of labor, and gender ideology. *Psychol. Sex. Orient. Gender Divers.* 4 438–450.

[B67] Taubman-Ben-Ari O. Spielman V. (2014). Personal growth following the first child’s birth: a comparison of parents of pre-and full-term babies. *Soc. Work Res.* 38 91–106. 10.1093/swr/svu011

[B68] Thompson A. D. Dodge D. (2018). *The Cost Of Becoming A Gay Dad: The Ultimate Guide.* Available online at: https://www.gayswithkids.com/the-cost-of-gay-becoming-a-gay-dad-2465969199.html?rebelltitem=1#rebelltitem1 (accessed October 16, 2018).

[B69] Tornello S. L. Sonnenberg B. N. Patterson C. J. (2015). Division of labor among gay fathers: associations with parent, couple, and child adjustment. *Psychol. Sex. Orient. Gender Divers.* 2 365–375. 10.1037/sgd0000109

[B70] Van Belle J. (2016). Paternity and parental leave policies across the European Union. *Eur. Comm.* Available online at: https://www.rand.org/pubs/research_reports/RR1666.html (accessed July 13, 2019).

[B71] Van Egeren L. A. (2004). The development of coparenting relationship over the transition to parenthood. *Infant Ment. Health J.* 25 453–477. 10.1002/imhj.20019

[B72] Van Ginkel J. R. (2010). *MI-MUL2.SPS (Computer Code).* Available online at: https://www.universiteitleiden.nl/en/staffmembers/joost-van-ginkel#tab-1 (accessed November 12, 2019).

[B73] Van Ginkel J. R. Kroonenberg P. M. (2014). Analysis of variance of multiply imputed data. *Multiv. Behav. Res.* 49 78–91. 10.1080/00273171.2013.855890 24860197 PMC4029775

[B74] Van Rijn - van Gelderen L. Bos H. W. M. Jorgensen T. D. Ellis-Davies K. Winstanley A. Golombok S. (2018). Wellbeing of gay fathers with children born through surrogacy: a comparison with lesbian-mother families and heterosexual IVF parent families. *Human Reprod*. 33 101–108. 10.1093/humrep/dex339 29145594

[B75] Vecho O. Gross M. Poteat V. (2011). Partage des tâches parentales au sein des couples de mères lesbiennes françaises ayant eu recours à une insémination artificielle avec donneur anonymedivision of parental labor in french lesbian mother families created by anonymous donor insemination. *Psychol. Franç.* 56 1–18. 10.1016/j.psfr.2010.11.002

[B76] West C. Zimmerman D. H. (1987). Doing gender. *Gender Soc.* 1 125–151.

[B77] Wu W. Jia F. Enders C. (2015). A comparison of imputation strategies for ordinal missing data on likert scale variables. *Multiv. Behav. Res.* 50 484–503. 10.1080/00273171.2015.1022644 26610248

