# Corrigendum: Determinants of Non-paid Task Division in Gay-, Lesbian-, and Heterosexual-Parent Families With Infants Conceived Using Artificial Reproductive Techniques

**DOI:** 10.3389/fpsyg.2020.01544

**Published:** 2020-07-16

**Authors:** Loes Van Rijn - Van Gelderen, Kate Ellis-Davies, Marijke Huijzer-Engbrenghof, Terrence D. Jorgensen, Martine Gross, Alice Winstanley, Berengere Rubio, Olivier Vecho, Michael E. Lamb, Henny M. W. Bos

**Affiliations:** ^1^Preventive Youth Care, Research Institute of Child Development and Education, University of Amsterdam, Amsterdam, Netherlands; ^2^Department of Psychology, Swansea University, Swansea, United Kingdom; ^3^Research Institute of Child Development and Education, University of Amsterdam, Amsterdam, Netherlands; ^4^UMR 8216 Centre d'Etudes en Sciences Sociales du Religieux, Paris, France; ^5^Division of Social and Developmental Psychology, University of Cambridge, Cambridge, United Kingdom; ^6^Institut Français des Sciences et Technologies des Transports, de l'Aménagement et des Réseaux, Paris, France; ^7^Département de Sciences Psychologiques, Université Paris Nanterre, Nanterre, France

**Keywords:** non-paid task division, parenting, gay, lesbian, heterosexual, first-time parents, infants, determinants

In the original article, we neglected to include the funder, the Jacobs Foundation, Grant: 015 11 63 00 to Kate Ellis-Davies.

The updated Funding section reads as follows:

This research was supported, under the auspices of the Open Research Area (Application BO 3973/1-1; Principal Investigator, ML), by grants from the UK Economic and Social Research Council (ESRC; Grant ES/K006150/1; Principal Investigator, ML), The Netherlands Organisation for Scientific Research (NWO; Grant NWO 464-11-001, Principal Investigator, HB), the French Agence Nationale de la Recherche (ANR; Grant ANR-12-ORAR-00005-01, Principal Investigator, OV), and the Jacobs Foundation (Grant 015 11 63 00 to KE-D) whose support is gratefully acknowledged.

The authors apologize for this error and state that this does not change the scientific conclusions of the article in any way. The original article has been updated.

